# Extent and Boundaries of Lymph Node Stations During Minimally Invasive Esophagectomy: A Survey Among Dutch Esophageal Surgeons

**DOI:** 10.1245/s10434-024-15475-7

**Published:** 2024-06-11

**Authors:** M. H. M. Ketel, D. C. van der Aa, S. P. G. Henckens, C. Rosman, M. I. van Berge Henegouwen, B. R. Klarenbeek, S. S. Gisbertz, DES Collaboration Group, Suzanne S. Gisbertz, Mark I. van Berge Henegouwen, Grard A. P. Nieuwenhuijzen, Misha D. P. Luyer, Bas P. L. Wijnhoven, Pieter van der Sluis, Peter van Duijvendijk, Edwin S. van der Zaag, Wobbe O. de Steur, Henk H. Hartgrink, Marloes Emous, Jean-Pierre E. N. Pierie, Johanna W. van Sandick, Koen J. Hartemink, Bastiaan R. Klarenbeek, Camiel Rosman, Stijn van Esser, Merlijn Hutteman, Jan Willem Haveman, Frederieke A. Dijkstra, Marc J. van Det, Ewout A. Kouwenhoven, Meindert N. Sosef, Eric H. J. Belgers

**Affiliations:** 1 Department of Surgery, Radboud University Medical Center, Nijmegen, The Netherlands; 2 Department of Surgery, Amsterdam UMC Location University of Amsterdam, Amsterdam, The Netherlands; 3 Cancer Center Amsterdam, Cancer Treatment and Quality of Life, Amsterdam, The Netherlands; 4 Department of Gastroenterology and Hepatology, Amsterdam Gastroenterology Endocrinology Metabolism, Amsterdam UMC location University of Amsterdam, Amsterdam, The Netherlands

**Keywords:** Esophagectomy, Lymphadenectomy, Lymph node dissection, Anatomical boundaries

## Abstract

**Background:**

The optimal extent of lymph node dissection (LND) and the anatomic boundaries per lymph node station (LNS) during minimally invasive esophagectomy (MIE) for esophageal cancer remain a topic of debate. This study investigated the opinion of Dutch esophageal cancer surgeons on their routine LND extent and anatomic boundaries per LNS during MIE.

**Methods:**

In April 2023, an English web-based cross-sectional survey was conducted. In each of the 15 Dutch hospitals performing MIE, two MIE surgeons were asked to participate. The routine LND extent (quantity, specific LNS) for distal esophageal adenocarcinoma, (dis)agreement with the TIGER definition, and anatomic boundaries for each LNS in six directions were queried.

**Results:**

The survey was completed by 24 Dutch MIE surgeons (80% response rate). Consensus on the routine LND extent ( ≥ 85% of the participating surgeons) included the left and right paracardial, left gastric artery, celiac trunk, proximal splenic artery, common hepatic artery, subcarinal middle mediastinal paraoesophageal, lower mediastinal paraoesophageal, pulmonary ligament, and upper mediastinal paraoesophageal LNSs. Other LNSs were not widely considered routine. Although, certain anatomic boundaries were consistent among the surgeons, the majority varied, even when they agreed on the TIGER definition.

**Conclusion:**

Significant variations in surgical practice among Dutch esophageal surgeons regarding their routine extent of LND and anatomic boundaries of LNSs during MIE were demonstrated. Variation may have an impact on clinical outcomes, hampering uniform treatment strategies and hindering comparison of performance assessments. This study highlighted the need for an international follow-up study toward one uniform defined LND during MIE for esophageal cancer.

**Supplementary Information:**

The online version contains supplementary material available at 10.1245/s10434-024-15475-7.

The incidence of esophageal cancer increases, annually resulting in 572.000 new cases, and with 508.600 estimated esophageal cancer-related deaths worldwide.^1^ In the Netherlands, the standard curative treatment for patients with a diagnosis of esophageal carcinoma involves a combination of neoadjuvant chemoradiotherapy (or perioperative chemotherapy) followed by an esophagectomy with two-field lymphadenectomy. Both the neoadjuvant chemoradiotherapy and lymph node dissection (LND) during esophagectomy have the potential to eliminate possible metastatic lymph nodes.^2^

To date, no consensus exists regarding the optimal routine LND.^3^ The Dutch national guideline does not specify the number of lymph node stations (LNSs) to be dissected,^4^ and although the Dutch Upper GI Cancer Audit (DUCA) uses the quality indicator of at least 15 dissected lymph nodes in the resection specimen,^5^ the locations of the LNSs are not specified. Besides, different classification systems to address lymph node metastases of esophageal cancer are used worldwide.^6^

To overcome this problem, Schuring et al.^6^ proposed one uniform TIGER-study classification system with defined anatomic landmarks based on the 11th edition of the ‘Japanese Classification of Esophageal Cancer’ (JES) and the 8th edition of the ‘American Joint Committee on Cancer/Union for International Cancer Control’ (AJCC/UICC) classification.^7^ Even when consensus has been reached on which LNSs to dissect (the extent of LND), it is expected that discrepancies remain in the definition of anatomic boundaries of each LNS. This became apparent during the development of the procedure-specific competency assessment tool (MIE-CAT) by Ketel et al.^8^ to assess the surgical performance of minimally invasive esophagectomy (MIE) and to determine where most discrepancies were present in assessing the LND phases during MIE. Only with consensus on the anatomic boundaries can the resection of each LNS be adequately assessed.

To enhance comparability of the LND among surgeons and institutions worldwide for research purposes and to generate uniform treatment strategies, explicit anatomic boundaries of each LNS, including both superior/inferior, ventral/dorsal, and left/right borders, are needed.

This study aimed to investigate the perspectives of Dutch esophageal surgeons regarding the extent of their LND and their anatomic boundaries per LNS during LND in MIE. This insight will be the essential foundation toward a consensus on the definition of a complete LND during MIE for esophageal cancer.

## Methods

### Study Design

This cross-sectional survey study among Dutch MIE surgeons was the first step in a larger study project toward consensus on the optimal routine extent of LND (specific stations to dissect) and the definition of anatomic boundaries per LNS during LND in MIE for esophageal cancer.

### Study Participants

A nationwide survey targeted esophageal surgeons in the Netherlands. From each of the 15 Dutch hospitals performing more than 20 (robot-assisted) MIEs per year according to the nationwide guideline,^4^ two surgeons were asked to participate. These surgeons were identified through previous esophageal surgery collaborations.^9^ In April 2023, a total of 30 Dutch esophageal cancer surgeons were invited to participate via extended e-mail invitations to participate in an English web-based survey. If the survey was not completed after 2 weeks, three subsequent reminders were sent (in April, May, and June) to encourage participation. Surgeons who did not respond or complete the survey after receiving these reminders (*n *= 6) were excluded.

### Survey, Data Collection, and Outcome Measures

The survey was created using Castor Electronic Data Capture and contained five sections (Table S1). First, the participants provided information related to their experience, routine approach, and techniques of MIE. Second, the surgeon’s routine extent of the abdominal and thoracic LND for distal esophageal adenocarcinoma was collected, based on both the TIGER^7^ and the Japanese Classification of Esophageal Cancer (JES) classification. If at least 85% of the participating surgeons routinely dissected an LNS, it was considered that general agreement had been reached and therefore part of the consensus-based routine LND. Third, anatomic boundaries according to the TIGER definition^7^ of each indicated routinely dissected LNSs were displayed, and (dis)agreement with this definition was asked. Additionally, surgeons had the opportunity to provide in-practice superior, inferior, ventral, dorsal, left, and right anatomic boundaries per LNS. Fourth, the relevance of a complete LND and factors influencing the in-practice anatomic boundaries per LNS in LND were questioned. Fifth, the origin of each surgeon’s point of view regarding the anatomic boundaries of the LNSs he or she used were subtracted.

Fully completed surveys were included for analyses, and the analyses were conducted anonymously. Data are presented as means with standard deviations (SDs) or as numbers with percentages (%). Statistical analysis was performed using IBM SPSS Statistics for Windows version 28.0 (IBM Corp., Orchard Road, Armonk, New York, USA).

## Results

### Surgeon Characteristics

In June 2023, 24 esophageal surgeons from 12 Dutch hospitals completed the survey (response rate, 80%). The characteristics of these surgeons are summarized in Table 1. Regarding surgical approach, the majority of the hospitals performed a transthoracic MIE (*n *= 23,95.8%), and an intrathoracic anastomosis was preferred in the majority of their cases (*n *= 20,83.3%). The average experience with MIE was 10 years, and the average date for starting MIE was 2013 (range, 2003–2019).

**Table 1:** Background questions regarding experience, routine approach, and techniques of MIE

	Surgeons
	(*n* = 24)
	*n* (%)
Primary MIE approach	
Transhiatal	1 (4.2)
Transthoracic	23 (95.8)
Primary anastomosis location for distal esophagus carcinoma	
Intrathoracic	20 (83.3)
Cervical	4 (16.7)
Primary anastomotic technique	
Circular end-to-side	6 (25.0)
Linear side-to-side	14 (58.3)
Hand-sewn end-to-end	4 (16.7)
Regular operation approach	
MIE	16 (66.7)
Robot-assisted MIE	6 (25.0)
Hybrid	1 (4.2)
Open	1 (4.2)
Type of hospital	
Academic	11 (45.8)
General teaching	13 (54.2)
Overall experience with MIE cases managed (including training)	
<40	1 (4.2)
40–80	1 (4.2)
80–120	2 (8.4)
>120	20 (83.3)
Mean year surgeons started performing MIE in hospital: year ± SD, (mininum–maximum)	2013 ± 4.0 (2003–2019)

MIE, minimally invasive esophagectomy

### Routine Extent of LND

**Fig. 1:**
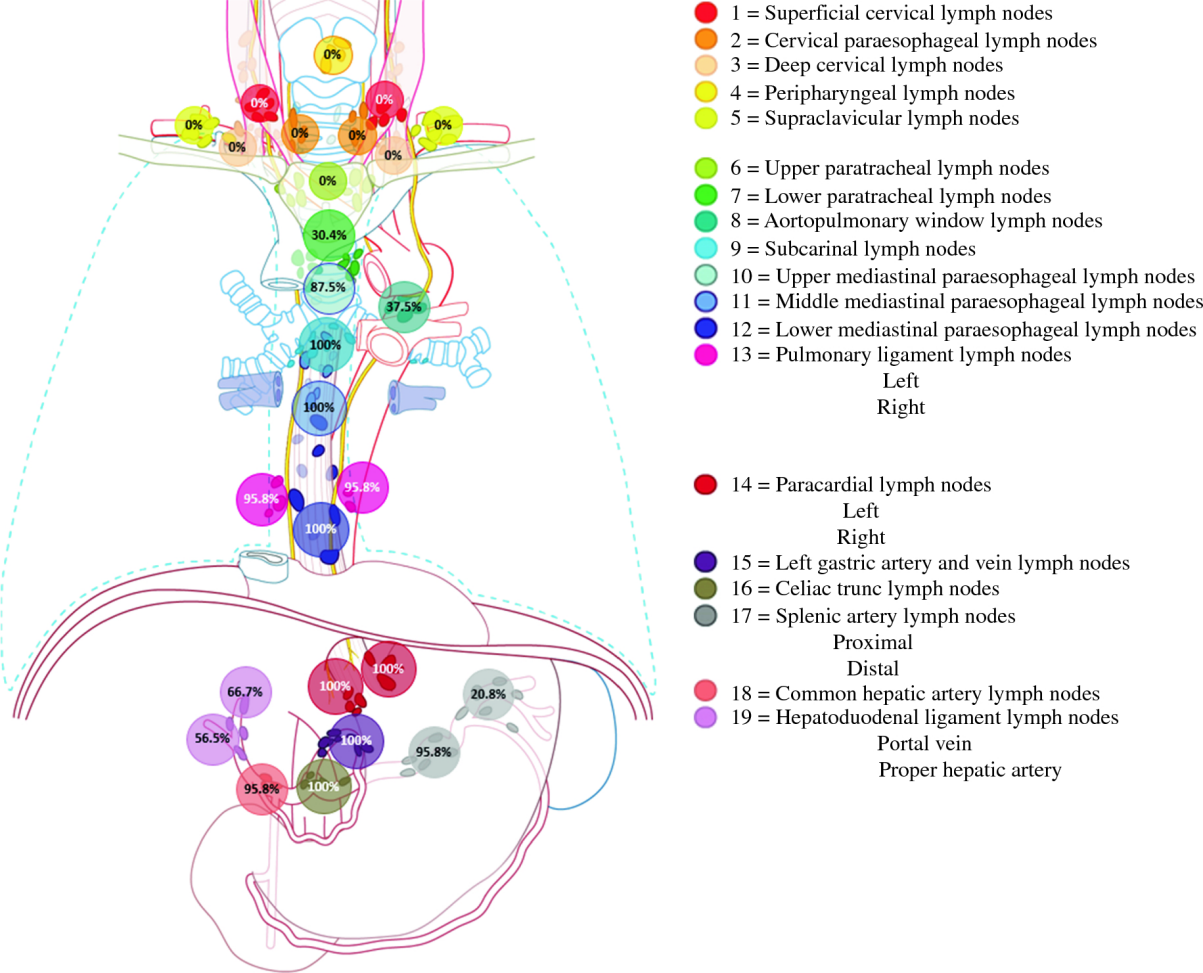
Routine extent of the lymph node dissection (LND) for distal esophageal adenocarcinoma. The percentage indicates the number of Dutch surgeons dissecting the stations. Adapted from the TIGER study protocol with permission of the authors.

The response regarding surgeons’ routine extent of abdominal and thoracic LND for distal esophageal adenocarcinoma is displayed in Fig. 1 and Table 2. All 24 surgeons (100%) agreed that the abdominal part of the LND should include at least the left and right paracardial, left gastric artery and vein, and celiac trunk stations. The majority also routinely dissected the proximal splenic artery (95.8%) and common hepatic artery lymph nodes (95.8%). All 24 surgeons (100%) agreed that LND in the thorax should include at least the subcarinal, middle, and lower mediastinal paraoesophageal LNSs, and most of the surgeons also routinely dissected the left and right pulmonary ligament (95.8%) as well as the upper mediastinal paraoesophageal (87.5%) lymph nodes. The LNSs not routinely dissected for a distal esophageal adenocarcinoma included the hepatoduodenal ligament (portal vein [66.7%], proper hepatic artery [56.5%]) and aortopulmonary window (37.5%), as well as the lower paratracheal (30.4%), distal splenic artery (20.8%), upper paratracheal (0%), and all cervical (0%) LNSs.

**Table 2:** Routine LND during MIE for distal adenocarcinoma

	Surgeons,	Difference within center	Agreement TIGER definition
	(*n* = 24)	(*n* = 12)	Yes (%)
	*n* (%)	*n* (%)	
Abdominal LNS			
Paracardial			
Right (#14R/JPN 1)	24 (100)	0	20 (83.3)
Left (#14L/JPN 2)	24 (100)	0	20 (83.3)
Left gastric artery and vein (#15/JPN 3a & 7)	24 (100)	0	21 (87.5)
Celiac trunk (#16/JPN 9)	24 (100)	0	22 (91.7)
Splenic artery			
Proximal (#17/JPN 11p)	23 (95.8)	1 (8.3)	22 (95.7)
Distal (#17/JPN 11d)	5 (20.8)	3 (25.0)	4 (80.0)
Common hepatic artery (#18/JPN 8a & p)	23 (95.8)	1 (8.3)	23 (100)
Hepatoduodenal ligament (#19)			
Portal vein (JPN 12p)	16 (66.7)	4 (33.3)	13 (81.2)
Proper hepatic artery (JPN 12a & 16)	13 (56.5)	5 (41.7)	10 (76.9)
Cervical LNS			
Superficial cervical (#1/JPN 100)	0	0	–
Cervical paraoesophageal (#2/JPN 101)	0	0	–
Deep cervical (#3/JPN 102)	0	0	–
Parapharyngeal (#4/JPN 103)	0	0	–
Supraclavicular (#5/JPN 104)	0	0	–
Thoracic LNS			
Upper paratracheal L/R (#6/JPN 106 pre & rec)	0	0	–
Lower paratracheal L/R (#7/JPN 106tb)	7 (30.4)	3 (25.0)	6 (85.7)
Aortopulmonary window (#8/JPN 113)	9 (37.5)	3 (25.0)	6 (66.7)
Subcarinal (#9/JPN 107 & 109)	24 (100)	0	22 (91.6)
Mediastinal paraoesophageal			
Upper (#10/JPN 105)	21 (87.5)	1 (8.3)	18 (85.7)
Middle (#11/JPN 108)	24 (100)	0	23 (95.8)
Lower (#12/JPN 110 & 111)	24 (100)	0	22 (91.6)
Pulmonary ligament nodes (#13/JPN 112 pul & oa)	23 (95.8)	1 (8.3)	-
Left (#13L)	–	–	19 (82.6)
Right (#13R)	–	–	21 (91.3)

LND, lymph node dissection; MIE, minimally invasive esophagectomy; LNS, lymph node station; JPN

Interestingly, within the 12 respondents’ centers, differences between surgeons of the same center were found regarding their routine extent of LND. These differences between surgeons of the same center concerned abdominal LNSs of the hepatoduodenal ligament (the portal vein [*n *= 4, 33.3%] and proper hepatic artery [*n *= 5, 41.7%]), distal splenic artery (*n *= 3, 25.0%), proximal splenic artery (*n *= 1, 8.3%), and common hepatic artery (*n *= 1, 8.3%). In the thorax, this concerned LNSs of the lower paratracheal lymph nodes (*n *= 3, 25.0%), aortopulmonary window lymph nodes (*n *= 3, 25.0%), upper mediastinal paraoesophageal lymph nodes (*n *= 1, 8.3%), and pulmonary ligament lymph nodes (*n *= 1, 8.3%).

### Influences on the Anatomic Boundaries and Relevance of the Boundaries

The surgeons indicated that the anatomic boundaries of each LNS (“100 percent complete dissection per LNS”; mean, 4.3 ± 0.6) were more relevant than the extent of the LND (“number of LNSs”; mean, 3.7 ± 0.9). Moreover, both the extent of LND and the anatomic boundaries per LNS were influenced mostly by patient factors (66.7% and 75%, respectively) and tumor location (79.2% and 62.5%, respectively), as shown in Table 3.

**Table 3:** Parameters influencing the extent of LND and anatomic boundaries

Factor	Extent of LND	Anatomic boundaries per LNS
	*n* (%)	
		*n* (%)
Patient and tumor factors (e.g., comorbidity, lymph node staging, tumor type)	16 (66.7)	18 (75.0)
Risk of morbidity	9 (37.5)	12 (50.0)
Possible survival benefit	10 (41.7)	10 (41.7)
Tumor location	19 (79.2)	15 (62.5)
Other	3 (not specified)	1 (none of the above)

LND, lymph node dissection; LNS, lymph node station

### Anatomic Boundaries

Tables 4 and 5 display the six directions of the thoracic and abdominal anatomic boundaries provided by the surgeons, together with the TIGER definitions of these stations. Between these anatomic boundaries, both consensus and differences were found. For example, for the majority, the ventral anatomic boundary of the celiac trunk LNS was the left gastric artery (× 11), and for some, it was the stomach (× 1), the overlying peritoneum (× 1), or the specimen (× 1). However, more distinctive differences were found for the inferior anatomic boundary of the celiac trunk LNS. These included the left gastric splenic and hepatic artery (× 5), the pancreas (× 4), and the celiac trunk (× 3). In addition, a significant number of anatomic boundary answers included “arbitrary” or “not applicable or no clearly described boundary.”

**Table 4:** Anatomical boundaries from the thoracic LNS provided by surgeons.

Thoracic stations	Alternative description	Superior	Inferior	Ventral	Dorsal	Left	Right
**Upper paratracheal (#6)** Boundaries provided by n = 0							
TIGER definition	Right: Lymph nodes located around the upper thoracic esophagus posterior to the right vagal nerve. Lymph nodes located along the anterior and lateral wall of the thoracic trachea until the level of the right vagal nerve. Lymph nodes located along the right recurrent laryngeal nerve in the mediastinum. The superior boundary is drawn from the cephalic border of the right subclavian artery to the suprasternal notch. Left: Lymph nodes located around the upper thoracic esophagus. Lymph nodes located along the anterior and lateral wall of the thoracic trachea until the upper margin of the aortic arch. Lymph nodes located along the left recurrent laryngeal nerve in the mediastinum. The superior boundary is drawn from the cephalic border of the left subclavian artery to the suprasternal notch.						
**Lower paratracheal (#7)** Boundaries provided by n = 3	To anterior side of **trachea** (1x)	Alongside distal **trachea**, incl. tracheobronchial angle (1x) **Brachiocephalic vein**/vagal nerve (1x) *NA (1x)*	Tracheobronchial angle (1x) **Bronchus** (1x)	Superior **vena cava** (1x) *NA (1x)*	**Spine** (1x) *NA (1x)*	**Trachea** (1x) *NA (1x)*	**Trachea** (1x) *NA (1x)*
TIGER definition	Right: Lymph nodes located in the tracheobronchial angle and located along the anterior and lateral wall of the thoracic trachea. The superior boundary is the vagal nerve, the ventral boundary the superior vena cava. Left: Lymph nodes located in the tracheobronchial angle and located along the anterior and lateral wall of the thoracic trachea. Lymph nodes located along the azygos vein arch and the right bronchial artery are included. Lymph nodes along the proximal part of the left recurrent laryngeal nerve along the aortic arch are also included. The superior boundary is the inferior wall of the aortic arch, and the lymph nodes are located in the area surrounded by the medial wall of the aortic arch.						
**Aortopulmonary window (#8)** Boundaries provided by n = 5	Aortic arch and left main bronchus (1x)	**Aortic arch** (4x)	Left (main) **bronchus** (3x) **Pulmonary artery** (1x)	**Pulmonary artery** (2x) *NA (2x)*	Left **main bronchus** (1x) **Vagal nerve** (1x) *NA (2x)*	*NA (4x)*	*NA (4x)*
**TIGER definition**	Subaortic and para-aortic nodes lateral to the arterial ligament. Superior boundary is the lower margin of the aortic arch. Ventral boundary is the pulmonary artery, distal boundary the left main bronchus.						
**Subcarinal (#9)** Boundaries provided by n = 11	Subcarinal area till upper side inferior pulmonary vein: carina (1x)	**Tracheal bifurcation**/carina (10x)	(Inferior) **pulmonary vein** (3x) (Upper) **para-esophageal lymph nodes** (2x) Visual most caudal border of lymph node tissue at level of carina (1x) Ends of main bronchi L&R (1x) *NA (5x)*	**Pericardium** (7x) **Pulmonary artery** (1x) *NA (3x)*	**Esophagus** (6x) **Fascia** **Aorta** *NA (3x)*	(Left) (**main**) **bronchus** (6x) Lateral margin of the **trachea** (2x) **Ligament** (1x) **Arbitrary**, distinction between hilar and subcarinal nodes sometimes not completely evident (1x) *NA (1x)*	(Right) (**main**) **bronchus** (6x) Lateral margin of the **trachea** (2x) **Ligament** (1x) **Arbitrary**, distinction between hilar and subcarinal nodes sometimes not completely evident (1x) *NA (1x)*
TIGER definition	Lymph nodes located caudal to the carina of the trachea. The lateral boundaries are the extended line of both lateral margins of the trachea						
**Upper mediastinal paraoesophageal (#10)** Boundaries provided by n = 6		**Thoracic aperture**/sternal notch/2-3 cm below subclavian/ start of 'angle hair' suggestive of entering cervical region (5x) **Pulmonary vein** (1x)	**Trachea bifurcation**/ upper border right and left bronchus (2x) **Subclavian artery** (1x) *NA (3x)*	Ventral/ membranous part of **trachea** (2x) **Thoracic wall** (1x) **Pericardium** (1x) *NA (2x)*	**Brachiocephalic** (1x) **Aorta** (1x) **Meso**-**esophagus** (1x) *NA (4x)*	Next to esophagus/**meso**-**esophagus** (2x) **Recurrent nerves** (1x) **Pulmonary ligament** (1x) *NA (2x)*	Next to esophagus/**meso**-**esophagus** (2x) **Recurrent nerves** (1x) **Lung** (1x) *NA (2x)*
TIGER definition	Dissection of the lymph nodes located around the upper thoracic esophagus. From the thoracic aperture until the trachea bifurcation.						
**Middle mediastinal paraoesophageal (#11)** Boundaries provided by n = 11		Tracheal bifurcation/**carina** (8x) **Subclavian artery** (1x) *NA (2x)*	(Upper border of/caudal margin of (inferior)) **pulmonary vein** (6x) **Lower** (esophageal) **lymph nodes** (2x) **Azygos vein** (1x) *NA (2x)*	(Trachea), **pericardium** (4x) **Superior caval vein** (1x) **Esophagus** (1x) **Aorta** (1x) *NA (4x)*	**Aorta** (3x) **Spine**/spinal column (2x) **Trachea** (1x) **Fascia** (1x) **Pericardium** (1x) All fatty tissue within **'meso-esophagus'** (1x) *NA (2x)*	**Lung**/pleura/ bronchus/pulmonary ligament (8x) All fatty tissue within **'meso-esophagus'**/ next to esophagus (2x) *NA (1x)*	**Lung**/pleura/ bronchus (6x) All fatty tissue within **‘meso-esophagus’**/(Next to) **esophagus** (3x) *NA (1x)*
TIGER definition	Lymph nodes located around the middle thoracic esophagus. From the tracheal bifurcation to the caudal margin of the inferior pulmonary vein						
**Lower mediastinal paraoesophageal (#12)** Boundaries provided by n = 11	These (academic) boundaries are not sharply delineated in (my) clinical practice (1x)	(Caudal margin of the) (inferior) **pulmonary vein** (5x) **Middle** (esophageal) **lymph nodes** (3x) **Subclavian artery** (1x) *NA (1x)*	**Diaphragm**/ hiatus/crus/ esophagogastric junction (8x) **Azygos vein** (1x) **Paracardial lymph nodes** (1x)	**Pericardium** (3x) **Superior caval vein** (1x) **Aorta/spinal cord** (1x) **Pleura** (1x) *NA (4x)*	**Aorta** (2x) **Trachea** (1x) **Spinal column** (1x) **Pericardium** (1x) All fatty tissue within **'meso-esophagus'** (1x) *NA (4x)*	**Lung**/pleura/ pulmonary ligament (5x) All fatty tissue within **'meso-esophagus'**/ next to esophagus (2x) *NA (3x)*	(Next to) **esophagus**/ all fatty tissue within ‘meso-esophagus’ (3x) **Lung**/pleura (4x) *NA (3x)*
TIGER definition	Lymph nodes located around the lower thoracic esophagus. From the caudal margin of the inferior pulmonary vein to the esophagogastric junction.						
**Right pulmonary ligament (#13R)** Boundaries provided by n = 8	These (academic) boundaries are not sharply delineated in (my) clinical practice (1x)	(Caudal margin of the) (inferior) **pulmonary vein** (5x) **Lung** (1x) *NA (1x)*	**Diaphragm**/crus (4x) Visceral **pleura** surface/**pulmonary ligament** (2x) *NA (1x)*	**Pericardium** (2x) Visceral **pleura** surface (1x) **Vena cava** (1x) *NA (3x)*	(Visceral) **pleura** (surface) (2x) **Aorta** (1x) *NA (4x)*	**Esophagus** (4x) **Pleura** (1x) *NA (2x)*	**Lung** (parenchyma)/ pleura/ **pulmonary ligament** (6x) *NA (2x)*
TIGER definition	Dissection of the lymph nodes within the right inferior pulmonary ligament.						
**Left pulmonary ligament (#13L)** Boundaries provided by n = 8	These (academic) boundaries are not sharply delineated in (my) clinical practice (1x) Left pleura only resected en bloc near the hiatus, more cranially the pleura stays in situ and the left pulmonary ligament is not seen (1x) *NA (1x)*	(Caudal margin of the) (inferior) **pulmonary vein** (3x) *NA (1x)*	**Crus** (2x) Visceral **pleura** surface (1x) *NA (1x)*	**Pericardium** (2x) Visceral **pleura** surface (1x) *NA (1x)*	(Visceral) **pleura** (surface) (2x) **Aorta** (1x) *NA (1x)*	Left **lung**/pleura (2x) **Esophagus** (1x) *NA (1x)*	Pleura/ **lung** parenchyma (3x) **Para-esophageal lymph nodes** (1x) *NA (1x)*
TIGER definition	Dissection of the lymph nodes within the left inferior pulmonary ligament.						

NA: not applicable or no clearly described boundary. Similar boundaries have been merged within these Tables to display the differences and similarities between the boundaries used by surgeons.

**Table 5:** Anatomical boundaries from the abdominal LNS provided by surgeons.

**Abdominal stations**	**Alternative description**	**Superior**	**Inferior**	**Ventral**	**Dorsal**	**Left**	**Right**
**Right paracardial (#14R)** Boundaries provided by n = 13	Not those along a branch of the left gastric artery (1x) (Immediately) adjacent to gastroesophageal junction right side (do not look separately at the first branch of the left subphrenic artery) (2x) Along esophagus (1x)	**Crus**/hiatus/diaphragm/**gastro-esophageal junction** (8x) (En bloc with/On the right side above the diaphragm 2-3 cm along the) **Esophagus** (2x) **Para-esophageal**/fatty tissue (2x)	(Descending branch of/first branch of ascending limb of) **left gastric artery** (5x) (En bloc with) **esophagus/stomach** (2x) **Celiac trunk** (1x) (right, 2-3 cm under the) **gastroesophageal junction** (1x) *NA (2x)*	(End of/All fatty tissue of/en bloc with silver of) **diaphragm** (5x) **Pericardium** (1x) **Lesser curvature** (1x) Apex **crus** (1x) *NA (2x)*	**Aorta** (5x) (Lower border of) **crus** (2x) **Lesser curvature** (1x) **Gastro-esophageal junction** (1x) *NA (2x)*	(En bloc with/halfway) **esophagus**/Gastric wall/along with specimen (7x) **Gastro-esophageal junction** (4x)	(Right border of) **crus**/(en bloc with silver of) diaphragm (6x) **Liver** (1x) (First branch of the ascending limb of) the **left gastric arte**ry (1x) **Vena cava inferior**/end of fatty tissue (1x) *NA (1x)*
TIGER definition	Located immediately adjacent to the gastroesophageal junction, including those along the first branch of ascending limb of the left gastric artery						
**Left paracardial (#14L)** Boundaries provided by n = 12	Not those along a branch of the left subphrenic artery (1x) (Immediately) adjacent to gastroesophageal junction left side (do not look separately at the first branch of the left subphrenic artery) (2x)	**Crus**/hiatus/diaphragm (6x) (En bloc with/ above the diaphragm along the) **esophagus** (2x) **Para-esophageal**/fatty tissue (2x) **Gastro-esophageal junction**/mediastinum (1x)	(First branch of) **short gastric artery** (2x) **Fundus**/stomach (3x) (En bloc with) **esophagus** (1x) (left 2-3 cm under the) **gastreso-phageal junction** (1x) (Esophagogastric branch of **left subphrenic artery** (1x) **Splenic artery** (1x) *NA (2x)*	(All fatty tissue/at hiatus en bloc silver of) **diaphragm** (4x) **Diaphragm** (1x) **Pericardium** (1x) Apex **crus** (1x) *NA (2x)*	**Aorta** (4x) (Lower border of) **Crus** (3x) **Retro-peritoneum** (1x) **Gastro-esophageal junction** (1x) *NA (1x)*	(Left border of) **Crus** (4x) (At hiatus en bloc silver of) **Diaphragm** (2x) (Junction-) **Fundus** (2x) (Esophagogastric branch of) **left subphrenic artery** (1x) **End of fatty tissue** (1x) *NA (1x)*	(En bloc with/halfway ) **esophagus**/specimen/stomach/gastric wall (7x) **Gastro-esophageal junction** (3x)
TIGER definition	Located immediately adjacent to the gastroesophageal junction, including those along the esophago-cardiac branch of the left subphrenic artery						
**Left gastric artery and vein (#15)**							
**Left gastric artery (#15A)** Boundaries provided by n = 11	Along all branches of the left gastric artery (1x) A specific delineation is not useful or clinically relevant since lymph node stations are connected (1x)	**Crus**/end of fatty tissue (4x) (Alongside/ (caudal border of) first ascending branch of) **left gastric artery** (3x) **Stomach** (1x) *NA (2x)*	((2cm from) Origin of/inferior peritoneum surface surrounding) **left gastric artery** at celiac trunk (7x) **Hepatic and splenic artery** (1x) **Pancreas** (2x) **Celiac trunk** (1x)	(Origin of/ (first branch of ascending limb of)) **left gastric artery** (3x) **Stomach** (2x) **Lesser omentum** included in resection (2x) (2cm from) **celiac trunk** (1x) *NA (1x)*	(Origin of/basis) **left gastric artery** (4x) **Celiac trunk** (3x) **Aorta** /crus (2x)	**Splenic artery** (2x) **Specimen** (1x) **Stomach**/lesser curvature (1x) **End of fatty tissue** (1x) *NA (4x)*	**Hepatic artery**/lymph nodes (2x) **Liver** S2/S3 (1x) **End of fatty tissue** (1x) *NA (5x)*
**Left gastric vein (#15V)** Boundaries provided by n = 8	En bloc with left gastric artery lymph nodes (1x)	**Crus** (3x) (Caudal border of first branch of ascending limb of) **left gastric artery** (2x) (Alongside) **left gastric vein** (1x) **Paracardial** (1x) *NA (1x)*	(Origin of/inferior peritoneum fat pad of) **left gastric vein** (2x) **Pancreas** (1x) **Hepatic and splenic artery** (1x) **Pancreas** (1x) **Celiac trunk** (1x)	With specimen/**stomach** (2x) (Origin of/ first ascending branch of) **left gastric artery** (2x) (2cm from) **Celiac trunk** (1x) **Fat around gastric vein** (1x) *NA (1x)*	(Origin of) **left gastric vein** (2x) (Base/origin of) **left gastric artery** (2x) **Aorta** (1x) **Celiac trunk** (1x) **Vena porta** (1x)	(En bloc with) **splenic artery** (1x) **Stomach**/lesser curvature (1x) *NA (4x)*	**Hepatic artery** (1x) *NA (5x)*
TIGER definition	Along the course of the left gastric artery. Superior boundary is the caudal border of the first branch of the ascending limb of the left gastric artery. Proximal boundary is the origin of the left gastric artery.						
**Celiac trunk (#16)** Boundaries provided by n = 14	At the division of the left gastric artery, splenic artery and the common hepatic artery (1x) Explore dorsal site of celiac trunk for lymphatic tissue, but not explicitly onto the wall of the aorta (1x)	(V-shape of) **crus**/hiatus/dorsal junction crus (6x) **Aorta** (2x) 2cm above **celiac trunk** (1x) inferior border of **left gastric artery** *NA (3x)*	(Origin of) **left gastric artery**, **splenic and** (inferior border of common) **hepatic artery** (5x) **Pancreas** (/portal vein) (4x) (origin of) **Celiac trunk** (3x)	(Origin of /along the) **left gastric artery** and vein (11x) **Stomach** (1x) **Specimen** (1x) Overlying **peritoneum** (1x)	(Not bare but solid plane covering) **aorta** (and crus) (10x) **Portal vein** (1x) (Origin of/ dorsal border of) **left gastric, splenic, and common hepatic artery** (2x) *NA (1x)*	**Splenic artery** (5x) (Medial side of the left ‘leg’ of) the **crus** (2x) **Adrenal gland** (1x) **Pancreas** (1x) (2cm left to) the **celiac trunk** (1x) *NA (3x)*	**Common hepatic artery** (5x) **Vena cava** (3x) (2cm right to) **Celiac trunk** (1x) (Medial side of the right ‘leg’ of) the **crus** (1x) *NA (3x)*
TIGER definition	Located around the celiac trunk. Dorsal boundary is the aorta; ventral boundary is the origin of the left gastric artery.						
**Splenic artery (#17)**							
**Splenic artery proximal (#17prox)** Boundaries provided by n = 11		(Left) **crus**/hiatus (5x) **Stomach** (2x) Edge of **splenic arter**y (1x) *NA (3x)*	(Unto the) **pancreas**/pancreatic rim (8x) **Splenic artery** (2x) **Celiac trunk** (1x)	**Pancreas** (3x) **Stomach** (3x) **Gastrosplenic ligament**/bursa omentalis/overlaying peritoneum (3x) *NA (2x)*	**Retro**-**peritoneum** (4x) **Adrenal gland**/Gerota’s fascia (2x) (bare) **Splenic artery** (3x) (Unto the) **pancreas** (1x) **End of fatty tissue** (1x)	**Halfway** (between origin & tail of/distal) **splenic artery** (5x) (Before hilus of) **spleen** (3x) **Halfway pancreatic tail**/rim/pancreas border (3x) (Dorsal) **gastric artery** (1x) (Origin of) **gastric artery** posterior (1x)	(Origin of) **splenic artery** (5x) **Celiac trunk** (4x) **Left gastric artery** (1x) **Crus** (1x) **End of fatty tissue** (1x)
**Splenic artery distal (#17dist)** Boundaries provided by n = 2		**Spleen**/**diaphragm** (1x)	**Pancreas** (1x)	**Pancreas** (1x)	**Pancreatic tail** (1x) **Diaphragm** (1x)	(Hilus of) the **spleen** (2x)	Halfway **splenic artery** (2x) **Left gastric artery** (1x)
TIGER definition	Lymph nodes from the origin of the splenic artery along its course alongside the pancreatic tail, including those adjacent to the splenic artery distal to the pancreatic tail, and those on the roots of the short gastric arteries and those along the left gastroepiploic artery proximal to its 1st gastric branch.						
**Common hepatic artery (#18)** Boundaries provided by n = 12		(Visualization of) **portal vein** (4x) (Alongside) **common hepatic artery**/start proper hepatic artery (2x) **Crus**/vena cava (1x) **Peritoneal surface** (1x) *NA (2x)*	**Pancreas** (head/rim/caudal to common hepatic artery (5x) (Caudal border of) **Common hepatic artery** (2x) **Gastroduodenal artery** (1x) **Crus and vena cava** (1x) *NA (1x)*	**Bursa/overlying peritoneum**/fatty tissue (around common hepatic artery) (5x) **Pancreas** (1x) *NA (4x)*	**Portal vein** (4x) (Dorsal border of) **common hepatic artery** (3x) **Pancreas** (2x) **Aorta** (1x)	(Origin of) **common hepatic artery**/**Celiac trunk**/**left gastric artery** (7x) *NA (4x)*	(Origin of/first 1-2 cm of) **Proper hepatic** artery/Until **division** of **common hepatic artery**/**Hepato**-**duodenal ligament**/(Branch of) **gastroduodenal** artery/**Right gastric** artery (11x) *NA (1x)*
TIGER definition	From the origin of the common hepatic artery along its course until the division into the gastroduodenal and proper hepatic artery.						
**Gastroduodenal Ligament (#19)**							
**Portal vein (#19)** Boundaries provided by n = 6	Along the portal vein, until the liver hilum (1x) Ventral and left of vena porta (1x) Hepato-duodenal ligament is the boundary (1x)	**Peritoneum** (on lymph nodes) (1x) **Diaphragm** (1x) **Common hepatic**/**proper hepatic artery** (1x) (Cranial border of) **portal vein** *NA (1x)*	**Portal vein** (3x) **Proper hepatic artery (**1x) **Common hepatic artery lymph nodes** (1x)	**Peritoneum** on lymph nodes (2x) **Common hepatic**/**proper hepatic artery** (1x) *NA (2x)*	(Ventral surface of) **portal vein** (2x) **Diaphragm**/retroperitoneum (1x) **Vena cava** (1x) *NA (1x)*	(Under) **hepatic artery** (1x) **Aorta** (1x) (Origin of) **left gastric artery** (1x) *NA (2x)*	**Liver** hilum/corner of segment 1 (2x) **Peritoneum** on lymph nodes (1x) **Proper hepatic artery** (1x) *NA (1x)*
TIGER definition	Along the proper hepatic artery and along the portal vein in the caudal half between the confluence of the right and left hepatic ducts and the upper border of the pancreas.						
**Proper hepatic (#19)** Boundaries provided by n = 6	Along the hepatic artery until the liver hilum (1x) Between liver hilum/right liver artery and gastroduodenal artery (1x)	**Portal vein** (1x) **Division of hepatic arteries** (1x) **Liver hilu**m (1x) *NA (1x)*	**Pancreatic** rim (1x) **Gastroduodenal artery** (1x) **Proper hepatic artery** (1x) **Bile duct** (1x)	First **1-2 cm anterior** (1x) **Peritoneum** on lymph nodes (1x) *NA (2x)*	**Portal vein** (2x) **Proper hepatic artery** (1x) *NA (1x)*	**Common hepatic artery** (3x) *NA (2x)*	**Liver** (hilum) (2x) **Common bile duct** (1x) *NA (2x)*
TIGER definition	Along the proper hepatic artery and along the portal vein in the caudal half between the confluence of the right and left hepatic ducts and the upper border of the pancreas.						

NA: not applicable or no clearly described boundary. Similar boundaries have been merged within these Tables to display the differences and similarities between the boundaries used by surgeon

**Fig. 2:**
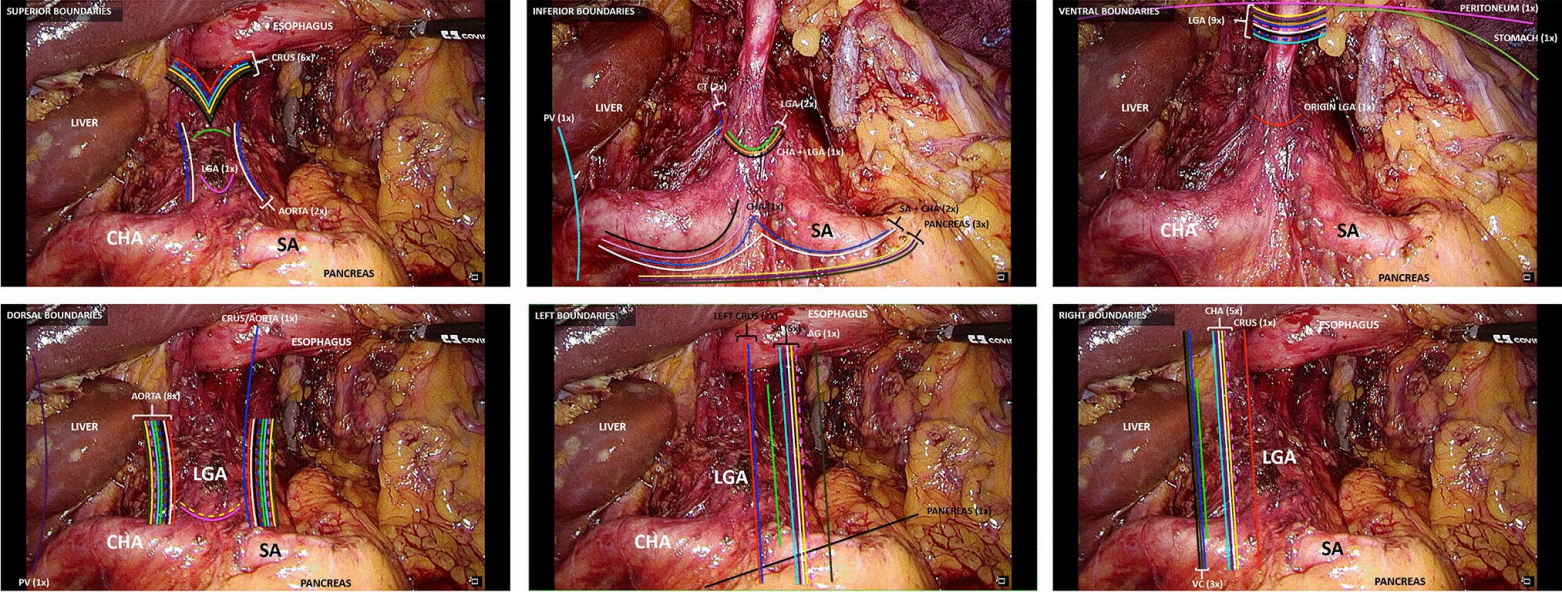
The anatomic boundaries of the celiac trunk lymph node station (LNS). Each colored line represents the survey results of a particular surgeon. (**A**) Superiod, (**B**) inferior, (**C**) ventral, (**D**) dorsal, (**E**) left, and (**F**) right, after ligation (**A, D, E,** and **F**) and before ligation (**B** and **C**). LGA, left gastric artery; CHA, common hepatic artery; SA, splenic artery; CT, celiac trunk; PV, portal vein; AG, adrenal gland; VC, vena cava

To illustrate similarities and differences in the provided anatomic boundaries, Fig. 2a–f shows a graphic representation of the variations regarding the celiac trunk LNS. Every line represents the anatomic boundaries provided by one individual surgeon, with corresponding colors of the surgeons between the Figures. Lines directly next to each other demonstrate the same anatomic boundary provided by the surgeons.

### Origin of Point of View

Overall, 20 surgeons (83.3%) indicated that they have changed their point of view regarding the extent (number of LNSs) and anatomic boundaries of their LND over time. Most (*n *= 11, 45.8%) found the extent more important over time, whereas others found the extent less important (*n *= 4, 16.7%). Moreover, other surgeons mentioned that the anatomic boundaries per LNS became more important (*n *= 4, 16.7%) or less important (*n *= 1, 4.2%). Furthermore, the surgeons indicated that both literature (*n *= 17, 70.8%) and sharing experiences with other surgeons/centers (*n *= 17, 70.8%) contributed most to their (changing) point of view regarding the anatomic boundaries of the LNSs, followed by training (*n *= 14, 58.3%), education (*n *= 13, 54.2%), own experience (*n *= 11, 45.8%), and congresses, symposia, and other meetings (*n *= 10, 41.7%).

## Discussion

This web-based survey confirmed the expected variability among Dutch esophageal surgeons with respect to both the extent of LND and the applied anatomic boundaries per LNS during MIE. Surgeons commonly presume that they have the same understanding of the stations and perform similar dissections. However, this study demonstrated this is not necessarily true. The observed variation brings challenges in current discussions regarding the benefit or harm of extended LNDs, and underscores the necessity of establishing consensus regarding the optimal routine LND for enhanced comparability in surgical patient care.

The Dutch surgeons indicated that they dissect at least 15 lymph nodes within the DUCA,^5^ but the specific lymph node stations dissected routinely remained unclear. This study showed nationwide consensus (≥ 85% of the participating Dutch esophageal surgeons) on the routine extent for distal esophageal adenocarcinoma encompassing the left and right paracardial, left gastric artery, celiac trunk, proximal splenic artery, common hepatic artery, subcarinal, upper mediastinal paraesophageal, middle mediastinal paraoesophageal, lower mediastinal paraesophageal, and pulmonary ligament LNSs (TIGER LNS 14, 15, 16, 17; proximal 18, 9, 10, 11, 12 and 13). This largely corresponds to the two-field or abdominal and mediastinal LND^3^ (except for the higher thoracic LNSs 6, 7 and 8 and the abdominal LNSs 17, distal, and 19.^7^ Surprisingly, the upper mediastinal paraoesophageal LNS (no. 10) was indicated to be routinely dissected by almost all the surgeons, with most of the surgeons performing Ivor-Lewis esophagectomy. Interestingly, discrepancies in the routine LND extent among the surgeons in the same hospital were noted for five participating hospitals. Although this seems remarkable, it confirms our hypothesis previously based on conversations and meetings with esophageal surgeons. Variability within a surgical team probably arises from the complexity of LND in esophagectomy and the lack of guidelines clearly delineating this. The observed heterogeneity in LND among surgeons is consistent with earlier findings^10^ and emphasizes the need for additional research toward consensus on the definition of complete LND.

The complexity of LND also appeared in the given answers when the surgeons were asked to provide anatomic boundaries for each LNS in six anatomic orientations. Even when indicating agreement with the TIGER definition, they provided anatomical boundaries for some stations that slightly deviated from the definition. Although consistency among surgeons was observed for certain stations in the description of anatomic boundaries per LNS (e.g., for 11 of 12 surgeons, the right border of the common hepatic artery LNS included the proper or common hepatic artery), the majority of LNSs showed (slight) variations (e.g., the inferior border of the common hepatic artery LNS included the pancreas [× 5], the common hepatic artery [× 2], the gastroduodenal artery [× 1], and the crus and vena cava [× 1]). The surgeons indicated that the anatomic boundaries they used were based primarily on literature and shared experiences among other surgeons in the field.^11^

The observed variations in the extent of the LND and the anatomic boundaries of each LNS hold significant clinical implications. These variations may explain the different outcomes in the existing literature. An extensive LND is currently advocated for enhancing tumor staging and potential long-term survival benefits,^2,12,13^ whereas there are concerns regarding the possible negative impact on morbidity and compromised quality of life due to excessive lymph node removal.^14^

Differences in anatomic boundaries hinder a comprehensive understanding of the precise components removed during the procedure. During dissection of the same LNSs, variations in boundaries may lead to diverse resected specimens and potentially result in distinct clinical outcomes for patients. It is anticipated that a clear understanding of anatomic boundaries will improve the comparability of LND among surgeons for research purposes. This includes comparison of performance assessments and their reliability among different reviewers^8^ as well as investigation of complications and survival benefits associated with LND, with the aim to generate uniform treatment strategies. Nevertheless, the observed variations in extent of LND and anatomic boundaries per LNS could serve as a foundation to establish consensus.

Our next step involves an international explorative study to investigate in-practice anatomic boundaries per LNS, incorporating assessments of short video clips of the LND with both the competency assessment tool^8^ and the surgical quality assessment tool.^15^ Research on this matter is indispensable because variation hinders comparing outcomes, and thereby valid assessments. Subsequently, we plan a Delphi study to achieve consensus on the LND extent and LNS anatomic boundaries.

This study was the first to investigate and describe the anatomic boundaries for the LND during MIE used by Dutch esophageal surgeons. However, it is important to acknowledge some limitations. First, describing the three-dimensional (3D) anatomic structure of LNSs in two-dimensional (2D) planes poses challenges. Although we selected this approach to provide a detailed and comprehensive overview, it may be worth exploring other visually representative methods that closely mirror clinical practice (e.g., drawing lines in a 3D anatomic model).

Second, described anatomic boundaries might differ from those of clinical practice because the dissection is based mostly on individual patient and tumor characteristics. For instance, the surgeon’s approach might be more radical, leading to removal of additional surrounding tissue beyond the currently defined boundaries. However, the surgeons were instructed to outline their anatomic boundaries for a “routine” LND as reference for the LND for distal adenocarcinoma, thereby eliminating the variation that tumor location could have in answers to the questions. Also, the vast majority of patients who undergo surgery for distal esophageal adenocarcinoma in the Netherlands present with stage cT3 and cN+ disease. Given the intermediate accuracy of current clinical tumor staging and the regular discordance between clinical and pathologic nodal staging, it may not be reliable enough to direct toward a certain extent of LND.^16^

Third, a complete overview of the current practices in the Netherlands was not established because three Dutch hospitals did not participate. Nevertheless, the insight of the 12 participating hospitals provided useful and valuable variations in LND extent and anatomic boundaries of LNSs.

Fourth, components of the web-based survey could have been described more clearly. Dividing the answer “patient and tumor factors” for the section discussing influences on the extent of the LND into “patient factors” and “tumor factors” might have yielded more comprehensive information. In addition, three LNSs (hepatoduodenal ligament, gastric artery, and splenic artery LNSs) received a more detailed description in the survey than in the TIGER definition. Because this could have caused some ambiguity in the description of the anatomic boundaries, these results were combined.

## Conclusion

This study demonstrated the routine extent and the exploration of anatomic boundaries for the LND during MIE, as performed by esophageal surgeons in the Netherlands. The lack of consensus on both the extent of the LND and the anatomic boundaries presents a challenge in gaining a comprehensive understanding of the precise removed components during the LND of MIE and complicates investigation of the survival benefits and risks of morbidity associated with LND. Further investigation is necessary to establish consensus regarding the anatomic boundaries for each LNS, and thereby the definition of a “complete” LND. These efforts will enhance comparability of LND practices among surgeons and institutions worldwide, facilitating the implementation of standardized treatment strategies and aiding research.

## Supplementary Information

Below is the link to the electronic supplementary material.

Supplementary file1 (PDF 4161 kb)

Supplementary file2 (DOCX 15 kb)
