# Correction: Extent and Boundaries of Lymph Node Stations During Minimally Invasive Esophagectomy: A Survey Among Dutch Esophageal Surgeons

**DOI:** 10.1245/s10434-025-18728-1

**Published:** 2025-11-23

**Authors:** M. H. M. Ketel, D. C. van der Aa, S. P. G. Henckens, C. Rosman, M. I. van Berge Henegouwen, B. R. Klarenbeek, S. S. Gisbertz, DES Collaboration Group, Suzanne S. Gisbertz, Mark I. van Berge Henegouwen, Grard A. P. Nieuwenhuijzen, Misha D. P. Luyer, Bas P. L. Wijnhoven, Pieter van der Sluis, Peter van Duijvendijk, Edwin S. van der Zaag, Wobbe O. de Steur, Henk H. Hartgrink, Marloes Emous, Jean-Pierre E. N. Pierie, Johanna W. van Sandick, Koen J. Hartemink, Bastiaan R. Klarenbeek, Camiel Rosman, Stijn van Esser, Merlijn Hutteman, Jan Willem Haveman, Frederieke A. Dijkstra, Marc J. van Det, Ewout A. Kouwenhoven, Meindert N. Sosef, Eric H. J. Belgers

**Affiliations:** 1 Department of Surgery, Radboud University Medical Center, Nijmegen, The Netherlands; 2 Department of Surgery, Amsterdam UMC Location University of Amsterdam, Amsterdam, The Netherlands; 3 Cancer Center Amsterdam, Cancer Treatment and Quality of Life, Amsterdam, The Netherlands; 4 Department of Gastroenterology and Hepatology, Amsterdam Gastroenterology Endocrinology Metabolism, Amsterdam UMC location University of Amsterdam, Amsterdam, The Netherlands

**Correction to: Annals of Surgical Oncology (2024) 31:5683-5696** 10.1245/s10434-024-15475-7

In the original online version of this article the following Acknowledgments were missing:

The collaborators of the DES Collaboration Group are as follows: Suzanne S. Gisbertz, Mark I. van Berge Henegouwen, Grard A.P. Nieuwenhuijzen, Misha D.P. Luyer, Bas P.L. Wijnhoven, Pieter van der Sluis, Peter van Duijvendijk, Edwin S. van der Zaag, Wobbe O. de Steur, Henk H. Hartgrink, Marloes Emous, Jean-Pierre E.N. Pierie, Johanna W. van Sandick, Koen J. Hartemink, Bastiaan R. Klarenbeek, Camiel Rosman, Stijn van Esser, Merlijn Hutteman, Jan Willem Haveman, Frederieke A. Dijkstra, Marc J. van Det, Ewout A. Kouwenhoven, Meindert N. Sosef, and Eric H.J. Belgers.

The original article was corrected.

