# Supplementary material for: Extent and Boundaries of Lymph Node Stations During Minimally Invasive Esophagectomy: A Survey Among Dutch Esophageal Surgeons
Source: Ann Surg Oncol. 2024 Jun 11;31(9):5683–96. doi: 10.1245/s10434-024-15475-7 (PMC11300550; doi:10.1245/s10434-024-15475-7)
Supplement: Supplementary file 1 — Supplementary file1 (PDF 4161 kb) [file 10434_2024_15475_MOESM1_ESM.pdf]

Survey 'Lymph node boundaries MIE'

Lymph node boundaries MIE - Background questions

Number	Question	Answers
1.1	What is your primary MIE approach?	◯ Transhiatal<br>◯ Transthoracic
1.2	What is your primary anastomosis location for distal esophageal cancer?	◯ Intrathoracic<br>◯ Cervical
1.3	What is your primary anastomosic technique?	◯ Circular End-to-Side<br>◯ Linear Side-to-Side<br>◯ Hand-sewn End-to-End<br>◯ Hand-sewn End-to-Side<br>◯ Other
1.3.1	***If 'What is your primary anastomosic technique?' is equal to 'Other' answer this question:***<br>Other anastomotic technique	
1.4	What is your regular operation approach?	◯ MIE (complete laparoscopic and thoracoscopic)<br>◯ RAMIE (complete robot-assisted)<br>◯ Hybrid (thorax open, abdominal lap or robotic)assisted (<br>◯ Hybrid (thoracoscopic or robotic, abdominal open)<br>◯ Open
1.5	What type of hospital is your center?	☐ Academic<br>☐ General teaching<br>☐ Non-teaching
1.6	What is your overall experience with MIE, including training years as fellow (cases)?	◯ <40<br>◯ 40-80<br>◯ 80-120<br>◯ >120
1.7	When did you start with MIE in your hospital?	

Lymph node boundaries MIE - Extent of lymphadenectomy

Number	Question	Answers
2.1	Abdominal lymph node stations	
2.2	What is your routine approach considering the *abdominal* lymph node dissection during esophagectomy for distal esophageal adenocarcinoma? (=beoogde lymfeklierdissectie)	☐ 14. Right paracardial LN (JES: 1)<br>☐ 14. Left paracardial LN (JES: 2)<br>☐ 15. Left gastric artery LN (JES: 3a&7)<br>☐ 15. Left gastric vein LN (JES: 3a&7)<br>☐ 16. Celiac trunk LN (JES: 9)<br>☐ 17. Proximal splenic artery LN (JES: 11p)<br>☐ 17. Distal splenic artery LN (JES: 11d)<br>☐ 18. Common hepatic artery LN (JES: 8a&8p)<br>☐ 19. Portal vein LN (JES: 12p)<br>☐ 19. Proper hepatic artery LN (JES: 12a&16)
2.3	Thoracic lymph node stations	
2.4	What is your routine approach considering the *thoracic* lymph node dissection during esophagectomy for distal esophageal adenocarcinoma? (=beoogde lymfeklierdissectie)	☐ 1. Superficial cervical lymph nodes (JES: 10)<br>☐ 2. Cervical paraoesophageal lymph nodes (JES: 101)<br>☐ 3. Deep cervical lymph nodes (JES: 102)<br>☐ 4. Peripharyngeal lymph nodes (JES: 103)<br>☐ 5. Supraclavicular lymph nodes (JES: 104)<br>☐ 6. Upper paratracheal lymph nodes (JES: 106pre&rec)<br>☐ 7. Lower paratracheal lymph nodes (JES: 106tb)<br>☐ 8. Aortapulmonary window lymph nodes (JES: 113)<br>☐ 9. Subcarinal lymph nodes (JES: 107&109)<br>☐ 10. Upper mediastinal paraoesophageal lymph nodes (JES: 105)<br>☐ 11. Middle mediastinal paraoesophageal lymph nodes (JES: 110&111)<br>☐ 12. Lower mediastinal paraoesophageal lymph nodes (JES: 112pulR)<br>☐ 13. Pulmonary ligament lymph nodes (JES: 112pulL&oa)

Lymph node boundaries MIE - Anatomical boundaries per lymph node station abdomen

Number	Question	Answers
3.1	Abdominal lymph node stations	

	To show the TIGER classification definition click on the 'i' icon on the left of the question	
3.2	***If 'What is your routine approach considering the abdominal lymph node dissection during esophagectomy for distal esophageal adenocarcinoma? (=beoogde lymfeklierdissectie)' is equal to '16. Celiac trunk LN (JES: 9)' answer this question:***<br>Celiac trunk: Do you use the definition of the TIGER study in practice for this lymph node station?	◯ Yes<br>◯ No
3.2.1	***If 'Celiac trunk: Do you use the definition of the TIGER study in practice for this lymph node station?' is equal to 'No' answer this question:***<br>If not, please provide a description of the definition you use for this lymph node station:	
3.3	***If 'What is your routine approach considering the abdominal lymph node dissection during esophagectomy for distal esophageal adenocarcinoma? (=beoogde lymfeklierdissectie)' is equal to '16. Celiac trunk LN (JES: 9)' answer this question:***<br>Please provide the anatomical boundaries you use 'in-practice' for this lymph node station	**In-practice used anatomical boundaries**<br>Superior<br>Inferior<br>Ventral<br>Dorsal<br>Left<br>Right
3.4	***If 'What is your routine approach considering the abdominal lymph node dissection during esophagectomy for distal esophageal adenocarcinoma? (=beoogde lymfeklierdissectie)' is equal to '17. Proximal splenic artery LN (JES: 11p)' answer this question:***<br>Proximal splenic artery: Do you use the definition of the TIGER study in practice for this lymph node station?	◯ Yes<br>◯ No
3.4.1	***If 'Proximal splenic artery: Do you use the definition of the TIGER study in practice for this lymph node station?' is equal to 'No' answer this question:***<br>If not, please provide a description of the definition you use for this lymph node station:	
3.5	***If 'What is your routine approach considering the abdominal lymph node dissection during esophagectomy for distal esophageal adenocarcinoma? (=beoogde lymfeklierdissectie)' is equal to '17. Proximal splenic artery LN (JES: 11p)' answer this question:***<br>Please provide the anatomical boundaries you use 'in-practice' for this lymph node station	**In-practice used anatomical boundaries**<br>Superior<br>Inferior<br>Ventral<br>Dorsal<br>Left<br>Right
3.6	***If 'What is your routine approach considering the abdominal lymph node dissection during esophagectomy for distal esophageal adenocarcinoma? (=beoogde lymfeklierdissectie)' is equal to '17. Distal splenic artery LN (JES: 11d)' answer this question:***<br>Distal splenic artery: Do you use the definition of the TIGER study in practice for this lymph node station?	◯ Yes<br>◯ No

3.6.1	***If 'Distal splenic artery: Do you use the definition of the TIGER study in practice for this lymph node station?' is equal to 'No' answer this question:***<br>If not, please provide a description of the definition you use for this lymph node station:	
3.7	***If 'What is your routine approach considering the abdominal lymph node dissection during esophagectomy for distal esophageal adenocarcinoma? (=beoogde lymfeklierdissectie)' is equal to '17. Distal splenic artery LN (JES: 11d)' answer this question:***<br>Please provide the anatomical boundaries you use 'in-practice' for this lymph node station	**In-practice used anatomical boundaries**<br>Superior<br>Inferior<br>Ventral<br>Dorsal<br>Left<br>Right
3.8	***If 'What is your routine approach considering the abdominal lymph node dissection during esophagectomy for distal esophageal adenocarcinoma? (=beoogde lymfeklierdissectie)' is equal to '14. Right paracardial LN (JES: 1)' answer this question:***<br>Right paracardial: Do you use the definition of the TIGER study in practice for this lymph node station?	○ Yes<br>○ No
3.8.1	***If 'Right paracardial: Do you use the definition of the TIGER study in practice for this lymph node station?' is equal to 'No' answer this question:***<br>If not, please provide a description of the definition you use for this lymph node station:	
3.9	***If 'What is your routine approach considering the abdominal lymph node dissection during esophagectomy for distal esophageal adenocarcinoma? (=beoogde lymfeklierdissectie)' is equal to '14. Right paracardial LN (JES: 1)' answer this question:***<br>Please provide the anatomical boundaries you use 'in-practice' for this lymph node station	**In-practice used anatomical boundaries**<br>Superior<br>Inferior<br>Ventral<br>Dorsal<br>Left<br>Right
3.10	***If 'What is your routine approach considering the abdominal lymph node dissection during esophagectomy for distal esophageal adenocarcinoma? (=beoogde lymfeklierdissectie)' is equal to '14. Left paracardial LN (JES: 2)' answer this question:***<br>Left paracardial: Do you use the definition of the TIGER study in practice for this lymph node station?	○ Yes<br>○ No
3.10.1	***If 'Left paracardial: Do you use the definition of the TIGER study in practice for this lymph node station?' is equal to 'No' answer this question:***<br>If not, please provide a description of the definition you use for this lymph node station:	

3.11	***If 'What is your routine approach considering the abdominal lymph node dissection during esophagectomy for distal esophageal adenocarcinoma? (=beoogde lymfeklierdissectie)' is equal to '14. Left paracardial LN (JES: 2)' answer this question:***<br>Please provide the anatomical boundaries you use 'in-practice' for this lymph node station	**In-practice used anatomical boundaries**<br>Superior<br>Inferior<br>Ventral<br>Dorsal<br>Left<br>Right
3.12	***If 'What is your routine approach considering the abdominal lymph node dissection during esophagectomy for distal esophageal adenocarcinoma? (=beoogde lymfeklierdissectie)' is equal to '15. Left gastric artery LN (JES: 3a&7)' answer this question:***<br>Left gastric artery: Do you use the definition of the TIGER study in practice for this lymph node station?	○ Yes<br>○ No
3.12.1	***If 'Left gastric artery: Do you use the definition of the TIGER study in practice for this lymph node station?' is equal to 'No' answer this question:***<br>If not, please provide a description of the definition you use for this lymph node station:	
3.13	***If 'What is your routine approach considering the abdominal lymph node dissection during esophagectomy for distal esophageal adenocarcinoma? (=beoogde lymfeklierdissectie)' is equal to '15. Left gastric artery LN (JES: 3a&7)' answer this question:***<br>Please provide the anatomical boundaries you use 'in-practice' for this lymph node station	**In-practice used anatomical boundaries**<br>Superior<br>Inferior<br>Ventral<br>Dorsal<br>Left<br>Right
3.14	***If 'What is your routine approach considering the abdominal lymph node dissection during esophagectomy for distal esophageal adenocarcinoma? (=beoogde lymfeklierdissectie)' is equal to '15. Left gastric vein LN (JES: 3a&7)' answer this question:***<br>Left gastric vein: Do you use the definition of the TIGER study in practice for this lymph node station?	○ Yes<br>○ No
3.14.1	***If 'Left gastric vein: Do you use the definition of the TIGER study in practice for this lymph node station?' is equal to 'No' answer this question:***<br>If not, please provide a description of the definition you use for this lymph node station:	

3.15	***If 'What is your routine approach considering the abdominal lymph node dissection during esophagectomy for distal esophageal adenocarcinoma? (=beoogde lymfeklierdissectie)' is equal to '15. Left gastric vein LN (JES: 3a&7)' answer this question:*** Please provide the anatomical boundaries you use 'in-practice' for this lymph node station	**In-practice used anatomical boundaries**<br>Superior<br>Inferior<br>Ventral<br>Dorsal<br>Left<br>Right
3.16	***If 'What is your routine approach considering the abdominal lymph node dissection during esophagectomy for distal esophageal adenocarcinoma? (=beoogde lymfeklierdissectie)' is equal to '18. Common hepatic artery LN (JES: 8a&8p)' answer this question:*** Common hepatic artery: Do you use the definition of the TIGER study in practice for this lymph node station?	○ Yes<br>○ No
3.16.1	***If 'Common hepatic artery: Do you use the definition of the TIGER study in practice for this lymph node station?' is equal to 'No' answer this question:*** If not, please provide a description of the definition you use for this lymph node station:	
3.17	***If 'What is your routine approach considering the abdominal lymph node dissection during esophagectomy for distal esophageal adenocarcinoma? (=beoogde lymfeklierdissectie)' is equal to '18. Common hepatic artery LN (JES: 8a&8p)' answer this question:*** Please provide the anatomical boundaries you use 'in-practice' for this lymph node station	**In-practice used anatomical boundaries**<br>Superior<br>Inferior<br>Ventral<br>Dorsal<br>Left<br>Right
3.18	***If 'What is your routine approach considering the abdominal lymph node dissection during esophagectomy for distal esophageal adenocarcinoma? (=beoogde lymfeklierdissectie)' is equal to '19. Portal vein LN (JES: 12p)' answer this question:*** Portal vein: Do you use the definition of the TIGER study in practice for this lymph node station?	○ Yes<br>○ No
3.18.1	***If 'Portal vein: Do you use the definition of the TIGER study in practice for this lymph node station?' is equal to 'No' answer this question:*** If not, please provide a description of the definition you use for this lymph node station:	

3.19	***If 'What is your routine approach considering the abdominal lymph node dissection during esophagectomy for distal esophageal adenocarcinoma? (=beoogde lymfeklierdissectie)' is equal to '19. Portal vein LN (JES: 12p)' answer this question:***<br>Please provide the anatomical boundaries you use 'in-practice' for this lymph node station	**In-practice used anatomical boundaries**<br>Superior<br>Inferior<br>Ventral<br>Dorsal<br>Left<br>Right
3.20	***If 'What is your routine approach considering the abdominal lymph node dissection during esophagectomy for distal esophageal adenocarcinoma? (=beoogde lymfeklierdissectie)' is equal to '19. Proper hepatic artery LN (JES: 12a&16)' answer this question:***<br>Proper hepatic artery: Do you use the definition of the TIGER study in practice for this lymph node station?	○ Yes<br>○ No
3.20.1	***If 'Proper hepatic artery: Do you use the definition of the TIGER study in practice for this lymph node station?' is equal to 'No' answer this question:***<br>If not, please provide a description of the definition you use for this lymph node station:	
3.21	***If 'What is your routine approach considering the abdominal lymph node dissection during esophagectomy for distal esophageal adenocarcinoma? (=beoogde lymfeklierdissectie)' is equal to '19. Proper hepatic artery LN (JES: 12a&16)' answer this question:***<br>Please provide the anatomical boundaries you use 'in-practice' for this lymph node station	**In-practice used anatomical boundaries**<br>Superior<br>Inferior<br>Ventral<br>Dorsal<br>Left<br>Right

Lymph node boundaries MIE - Anatomical boundaries per lymph node station thorax

Number	Question	Answers
4.1	Thoracic lymph node stations	
	To show the TIGER classification definition click on the 'i' icon on the left of the question	
4.2	***If 'What is your routine approach considering the thoracic lymph node dissection during esophagectomy for distal esophageal adenocarcinoma? (=beoogde lymfeklierdissectie)' is equal to '10. Upper mediastinal paraoesophageal lymph nodes (JES: 105)' answer this question:***<br>Right&Left paraesophageal upper mediastinal: Do you use the definition of the TIGER study in practice for this lymph node station?	○ Yes<br>○ No

4.2.1 ***If 'Right&Left paraesophageal upper mediastinal: Do you use the definition of the TIGER study in practice for this lymph node station?' is equal to 'No' answer this question:***
If not, please provide a description of the definition you use for this lymph node station:

4.3 ***If 'What is your routine approach considering the thoracic lymph node dissection during esophagectomy for distal esophageal adenocarcinoma? (=beoogde lymfeklierdissectie)' is equal to '10. Upper mediastinal paraoesophageal lymph nodes (JES: 105)' answer this question:***
Please provide the anatomical boundaries you use 'in-practice' for this lymph node station

	**In-practice used anatomical boundaries**
Superior	
Inferior	
Ventral	
Dorsal	
Left	
Right	

4.4 ***If 'What is your routine approach considering the thoracic lymph node dissection during esophagectomy for distal esophageal adenocarcinoma? (=beoogde lymfeklierdissectie)' is equal to '11. Middle mediastinal paraoesophageal lymph nodes (JES: 110&111)' answer this question:***
Right&Left paraesophageal middle mediastinal: Do you use the definition of the TIGER study in practice for this lymph node station?

○ Yes
○ No

4.4.1 ***If 'Right&Left paraesophageal middle mediastinal: Do you use the definition of the TIGER study in practice for this lymph node station?' is equal to 'No' answer this question:***
If not, please provide a description of the definition you use for this lymph node station:

4.5 ***If 'What is your routine approach considering the thoracic lymph node dissection during esophagectomy for distal esophageal adenocarcinoma? (=beoogde lymfeklierdissectie)' is equal to '11. Middle mediastinal paraoesophageal lymph nodes (JES: 110&111)' answer this question:***
Please provide the anatomical boundaries you use 'in-practice' for this lymph node station

	**In-practice used anatomical boundaries**
Superior	
Inferior	
Ventral	
Dorsal	
Left	
Right	

4.6 ***If 'What is your routine approach considering the thoracic lymph node dissection during esophagectomy for distal esophageal adenocarcinoma? (=beoogde lymfeklierdissectie)' is equal to '12. Lower mediastinal paraoesophageal lymph nodes (JES: 112pulR)' answer this question:***
Right&Left paraesophageal lower mediastinal: Do you use the definition of the TIGER study in practice for this lymph node station?

○ Yes
○ No

4.6.1	***If 'Right&Left paraesophageal lower mediastinal: Do you use the definition of the TIGER study in practice for this lymph node station?' is equal to 'No' answer this question:***<br>If not, please provide a description of the definition you use for this lymph node station:	
4.7	***If 'What is your routine approach considering the thoracic lymph node dissection during esophagectomy for distal esophageal adenocarcinoma? (=beoogde lymfeklierdissectie)' is equal to '12. Lower mediastinal paraoesophageal lymph nodes (JES: 112pulR)' answer this question:***<br>Please provide the anatomical boundaries you use 'in-practice' for this lymph node station	**In-practice used anatomical boundaries**<br>Superior<br>Inferior<br>Ventral<br>Dorsal<br>Left<br>Right
4.8	***If 'What is your routine approach considering the thoracic lymph node dissection during esophagectomy for distal esophageal adenocarcinoma? (=beoogde lymfeklierdissectie)' is equal to '13. Pulmonary ligament lymph nodes (JES: 112pulL&oa)' answer this question:***<br>Right pulmonary ligament: Do you use the definition of the TIGER study in practice for this lymph node station?	◯ Yes<br>◯ No
4.8.1	***If 'Right pulmonary ligament: Do you use the definition of the TIGER study in practice for this lymph node station?' is equal to 'No' answer this question:***<br>If not, please provide a description of the definition you use for this lymph node station:	
4.9	***If 'What is your routine approach considering the thoracic lymph node dissection during esophagectomy for distal esophageal adenocarcinoma? (=beoogde lymfeklierdissectie)' is equal to '13. Pulmonary ligament lymph nodes (JES: 112pulL&oa)' answer this question:***<br>Please provide the anatomical boundaries you use 'in-practice' for this lymph node station	**In-practice used anatomical boundaries**<br>Superior<br>Inferior<br>Ventral<br>Dorsal<br>Left<br>Right
4.10	***If 'What is your routine approach considering the thoracic lymph node dissection during esophagectomy for distal esophageal adenocarcinoma? (=beoogde lymfeklierdissectie)' is equal to '13. Pulmonary ligament lymph nodes (JES: 112pulL&oa)' answer this question:***<br>Left pulmonary ligament: Do you use the definition of the TIGER study in practice for this lymph node station?	◯ Yes<br>◯ No
4.10.1	***If 'Left pulmonary ligament: Do you use the definition of the TIGER study in practice for this lymph node station?' is equal to 'No' answer this question:***<br>If not, please provide a description of the definition you use for this lymph node station:	

4.11	***If 'What is your routine approach considering the thoracic lymph node dissection during esophagectomy for distal esophageal adenocarcinoma? (=beoogde lymfeklierdissectie)' is equal to '13. Pulmonary ligament lymph nodes (JES: 112pulL&oa)' answer this question:*** Please provide the anatomical boundaries you use 'in-practice' for this lymph node station	**In-practice used anatomical boundaries**<br>Superior<br>Inferior<br>Ventral<br>Dorsal<br>Left<br>Right
4.12	***If 'What is your routine approach considering the thoracic lymph node dissection during esophagectomy for distal esophageal adenocarcinoma? (=beoogde lymfeklierdissectie)' is equal to '1. Superficial cervical lymph nodes (JES: 10)' answer this question:*** Super ficial cervical: Do you use the definition of the TIGER study in practice for this lymph node station?	◯ Yes<br>◯ No
4.12.1	***If 'Super ficial cervical: Do you use the definition of the TIGER study in practice for this lymph node station?' is equal to 'No' answer this question:*** If not, please provide a description of the definition you use for this lymph node station:	
4.13	***If 'What is your routine approach considering the thoracic lymph node dissection during esophagectomy for distal esophageal adenocarcinoma? (=beoogde lymfeklierdissectie)' is equal to '1. Superficial cervical lymph nodes (JES: 10)' answer this question:*** Please provide the anatomical boundaries you use 'in-practice' for this lymph node station	**In-practice used anatomical boundaries**<br>Superior<br>Inferior<br>Ventral<br>Dorsal<br>Left<br>Right
4.14	***If 'What is your routine approach considering the thoracic lymph node dissection during esophagectomy for distal esophageal adenocarcinoma? (=beoogde lymfeklierdissectie)' is equal to '2. Cervical paraoesophageal lymph nodes (JES: 101)' answer this question:*** Cervical paraoesphageal: Do you use the definition of the TIGER study in practice for this lymph node station?	◯ Yes<br>◯ No
4.14.1	***If 'Cervical paraoesphageal: Do you use the definition of the TIGER study in practice for this lymph node station?' is equal to 'No' answer this question:*** If not, please provide a description of the definition you use for this lymph node station:	

4.15	***If 'What is your routine approach considering the thoracic lymph node dissection during esophagectomy for distal esophageal adenocarcinoma? (=beoogde lymfeklierdissectie)' is equal to '2. Cervical paraoesophageal lymph nodes (JES: 101)' answer this question:***<br>Please provide the anatomical boundaries you use 'in-practice' for this lymph node station	**In-practice used anatomical boundaries**<br>Superior<br>Inferior<br>Ventral<br>Dorsal<br>Left<br>Right
4.16	***If 'What is your routine approach considering the thoracic lymph node dissection during esophagectomy for distal esophageal adenocarcinoma? (=beoogde lymfeklierdissectie)' is equal to '3. Deep cervical lymph nodes (JES: 102)' answer this question:***<br>Deep cervical: Do you use the definition of the TIGER study in practice for this lymph node station?	○ Yes<br>○ No
4.16.1	***If 'Deep cervical: Do you use the definition of the TIGER study in practice for this lymph node station?' is equal to 'No' answer this question:***<br>If not, please provide a description of the definition you use for this lymph node station:	
4.17	***If 'What is your routine approach considering the thoracic lymph node dissection during esophagectomy for distal esophageal adenocarcinoma? (=beoogde lymfeklierdissectie)' is equal to '3. Deep cervical lymph nodes (JES: 102)' answer this question:***<br>Please provide the anatomical boundaries you use 'in-practice' for this lymph node station	**In-practice used anatomical boundaries**<br>Superior<br>Inferior<br>Ventral<br>Dorsal<br>Left<br>Right
4.18	***If 'What is your routine approach considering the thoracic lymph node dissection during esophagectomy for distal esophageal adenocarcinoma? (=beoogde lymfeklierdissectie)' is equal to '4. Peripharyngeal lymph nodes (JES: 103)' answer this question:***<br>Peripharyngeal: Do you use the definition of the TIGER study in practice for this lymph node station?	○ Yes<br>○ No
4.18.1	***If 'Peripharyngeal: Do you use the definition of the TIGER study in practice for this lymph node station?' is equal to 'No' answer this question:***<br>If not, please provide a description of the definition you use for this lymph node station:	

4.19 ***If 'What is your routine approach considering the thoracic lymph node dissection during esophagectomy for distal esophageal adenocarcinoma? (=beoogde lymfeklierdissectie)' is equal to '4. Peripharyngeal lymph nodes (JES: 103)' answer this question:***
Please provide the anatomical boundaries you use 'in-practice' for this lymph node station

	**In-practice used anatomical boundaries**
Superior	
Inferior	
Ventral	
Dorsal	
Left	
Right	

4.20 ***If 'What is your routine approach considering the thoracic lymph node dissection during esophagectomy for distal esophageal adenocarcinoma? (=beoogde lymfeklierdissectie)' is equal to '5. Supraclavicular lymph nodes (JES: 104)' answer this question:***
Supraclavicular: Do you use the definition of the TIGER study in practice for this lymph node station?

○ Yes
○ No

4.20.1 ***If 'Supraclavicular: Do you use the definition of the TIGER study in practice for this lymph node station?' is equal to 'No' answer this question:***
If not, please provide a description of the definition you use for this lymph node station:

4.21 ***If 'What is your routine approach considering the thoracic lymph node dissection during esophagectomy for distal esophageal adenocarcinoma? (=beoogde lymfeklierdissectie)' is equal to '5. Supraclavicular lymph nodes (JES: 104)' answer this question:***
Please provide the anatomical boundaries you use 'in-practice' for this lymph node station

	**In-practice used anatomical boundaries**
Superior	
Inferior	
Ventral	
Dorsal	
Left	
Right	

4.22 ***If 'What is your routine approach considering the thoracic lymph node dissection during esophagectomy for distal esophageal adenocarcinoma? (=beoogde lymfeklierdissectie)' is equal to '6. Upper paratracheal lymph nodes (JES: 106pre&rec)' answer this question:***
Right&Left upper paratracheal: Do you use the definition of the TIGER study in practice for this lymph node station?

○ Yes
○ No

4.22.1 ***If 'Right&Left upper paratracheal: Do you use the definition of the TIGER study in practice for this lymph node station?' is equal to 'No' answer this question:***
If not, please provide a description of the definition you use for this lymph node station:

4.23	***If 'What is your routine approach considering the thoracic lymph node dissection during esophagectomy for distal esophageal adenocarcinoma? (=beoogde lymfeklierdissectie)' is equal to '6. Upper paratracheal lymph nodes (JES: 106pre&rec)' answer this question:*** Please provide the anatomical boundaries you use 'in-practice' for this lymph node station	**In-practice used anatomical boundaries**<br>Superior<br>Inferior<br>Ventral<br>Dorsal<br>Left<br>Right
4.24	***If 'What is your routine approach considering the thoracic lymph node dissection during esophagectomy for distal esophageal adenocarcinoma? (=beoogde lymfeklierdissectie)' is equal to '7. Lower paratracheal lymph nodes (JES: 106tb)' answer this question:*** Right&Left lower paratracheal: Do you use the definition of the TIGER study in practice for this lymph node station?	○ Yes<br>○ No
4.24.1	***If 'Right&Left lower paratracheal: Do you use the definition of the TIGER study in practice for this lymph node station?' is equal to 'No' answer this question:*** If not, please provide a description of the definition you use for this lymph node station:	
4.25	***If 'What is your routine approach considering the thoracic lymph node dissection during esophagectomy for distal esophageal adenocarcinoma? (=beoogde lymfeklierdissectie)' is equal to '7. Lower paratracheal lymph nodes (JES: 106tb)' answer this question:*** Please provide the anatomical boundaries you use 'in-practice' for this lymph node station	**In-practice used anatomical boundaries**<br>Superior<br>Inferior<br>Ventral<br>Dorsal<br>Left<br>Right
4.26	***If 'What is your routine approach considering the thoracic lymph node dissection during esophagectomy for distal esophageal adenocarcinoma? (=beoogde lymfeklierdissectie)' is equal to '8. Aortapulmonary window lymph nodes (JES: 113)' answer this question:*** Aortopulmonary window: Do you use the definition of the TIGER study in practice for this lymph node station?	○ Yes<br>○ No
4.26.1	***If 'Aortopulmonary window: Do you use the definition of the TIGER study in practice for this lymph node station?' is equal to 'No' answer this question:*** If not, please provide a description of the definition you use for this lymph node station:	

4.27	***If 'What is your routine approach considering the thoracic lymph node dissection during esophagectomy for distal esophageal adenocarcinoma? (=beoogde lymfeklierdissectie)' is equal to '8. Aortapulmonary window lymph nodes (JES: 113)' answer this question:*** Please provide the anatomical boundaries you use 'in-practice' for this lymph node station	**In-practice used anatomical boundaries**<br>Superior<br>Inferior<br>Ventral<br>Dorsal<br>Left<br>Right
4.28	***If 'What is your routine approach considering the thoracic lymph node dissection during esophagectomy for distal esophageal adenocarcinoma? (=beoogde lymfeklierdissectie)' is equal to '9. Subcarinal lymph nodes (JES: 107&109)' answer this question:*** Subcarinal: Do you use the definition of the TIGER study in practice for this lymph node station?	○ Yes<br>○ No
4.28.1	***If 'Subcarinal: Do you use the definition of the TIGER study in practice for this lymph node station?' is equal to 'No' answer this question:*** If not, please provide a description of the definition you use for this lymph node station:	
4.29	***If 'What is your routine approach considering the thoracic lymph node dissection during esophagectomy for distal esophageal adenocarcinoma? (=beoogde lymfeklierdissectie)' is equal to '9. Subcarinal lymph nodes (JES: 107&109)' answer this question:*** Please provide the anatomical boundaries you use 'in-practice' for this lymph node station	**In-practice used anatomical boundaries**<br>Superior<br>Inferior<br>Ventral<br>Dorsal<br>Left<br>Right

Lymph node boundaries MIE - Influences on in practice completeness of the lymphadenectomy

Number	Question	Answers
5.1	How relevant do you find a 100% complete dissection per lymph node station (=radicality per lymph node station)?	Irrelevant (1) ... Very relevant (5)
5.2	What influences your intendend dissection completeness per lymph node station?	☐ Patient factors (e.g. staging of the tumor and/or lymph node stations)<br>☐ Chance on morbidity<br>☐ Chance of survival<br>☐ Tumor location<br>☐ Other

5.2.1	***If 'What influences your intendend dissection completeness per lymph node station?' is equal to 'Other' answer this question:***<br>Other	
5.3	How relevant do you find the extent of the lymph node dissection (= the amount of dissected lymph node stations)?	Irrelevant (1) … Very relevant (5)
5.4	What influences your intendend extensiveness of the lymph node dissection (= amount of lymph node stations to dissect)?	☐ Patient factors (e.g. staging of the tumor and/or lymph node stations)<br>☐ Chance on morbidity<br>☐ Chance of survival<br>☐ Tumor location<br>☐ Other
5.4.1	***If 'What influences your intendend extensiveness of the lymph node dissection (= amount of lymph node stations to dissect)?' is equal to 'Other' answer this question:***<br>Other	

Lymph node boundaries MIE - Origin of each surgeon's point of view

Number	Question	Answers
6.1	Which factors contributed to (changing) your point of view regarding the anatomical boundaries of the lymph node dissection?	☐ Education<br>☐ Training<br>☐ Literature<br>☐ Congressess, symposia, other meetings<br>☐ Own experience<br>☐ Sharing experiences with other surgeons/centers<br>☐ Other
6.1.1	***If 'Which factors contributed to (changing) your point of view regarding the anatomical boundaries of the lymph node dissection?' is equal to 'Other' answer this question:***<br>Other	
6.2	Did your point of view regarding the extent (# of lymph node stations) and anatomical boundaries of the lymph node dissection chance over time?	◯ Yes<br>◯ No

6.2.1	***If*** *'Did your po i t of v ew rega r g t e e tent* ***(#*** *of l ph node sta t ons) and anatomical bounda r es of t e lymph node d sec t on chance o v r t e?' i equal to* ***'Yes'*** *answer th i question:*<br>If yes, how did your point of view change?	◯ I find the radicality per lymph node station more important<br>◯ I find the radicality per lymph node station less important<br>◯ I find the extent (= amount of lymph node stations) more important<br>◯ I find the extent (= amount of lymph node stations) less important<br>◯ Other
6.2.1.1	*If ' I y s, how d d your po i t of view change?' is equal to 'Other' answer th i ques t on:*<br>Other	
