# Supplementary material for: Extent and Boundaries of Lymph Node Stations During Minimally Invasive Esophagectomy: A Survey Among Dutch Esophageal Surgeons
Source: Ann Surg Oncol. 2024 Jun 11;31(9):5683–96. doi: 10.1245/s10434-024-15475-7 (PMC11300550; doi:10.1245/s10434-024-15475-7)
Supplement: Supplementary file 2 — Supplementary file2 (DOCX 15 kb) [file 10434_2024_15475_MOESM2_ESM.docx]

**Supplementary B – Individual anatomical boundaries of the celiac trunk LNS, corresponding Figures 2a to 2f**

|  | Superior (fig.2a) | Inferior (fig.2b) | Ventral (fig.2c) | Dorsal (fig.2d) | Left (fig.2e) | Right (fig.2f) |
| --- | --- | --- | --- | --- | --- | --- |
| 1 Interrupted pink |  |  | left gastric artery | aorta | splenic artery | common hepatic artery |
| 2 Yellow | NA | pancreas | left gastric artery | aorta | splenic artery | common hepatic artery |
| 3 Dark purple | crus | pancreas | left gastric artery | portal vein | splenic artery | common hepatic artery |
| 4 Dark green | hiatus | pancreas | left gastric artery | aorta | adrenal gland | Vena cava |
| 5 Red | crus | celiac artery | origin of the left gastric artery | aorta | medial side of the left 'leg' of the crus | medial side of the right 'leg' of the crus |
| 6 Blue | aorta | whale’s tale | along the left gastric artery | aorta and crus | left crus | Vena cava |
| 7 Green | 2 cm above celiac trunk | left gastric artery | stomach | aorta | 2 cm left to celiac trunk | 2 cm right to celiac trunk |
| 8 Orange | gut feeling | origin of the left gastric artery | left gastric artery | NA | NA | NA |
| 9 Interrupted blue | dorsal junction of crura | origin of celiac trunk | left gastric artery | aorta | splenic nodes (en-bloc) | common hepatic nodes (en-bloc) |
